# Pro-inflammatory cytokine/chemokine production by reovirus treated melanoma cells is PKR/NF-κB mediated and supports innate and adaptive anti-tumour immune priming

**DOI:** 10.1186/1476-4598-10-20

**Published:** 2011-02-21

**Authors:** Lynette Steele, Fiona Errington, Robin Prestwich, Elizabeth Ilett, Kevin Harrington, Hardev Pandha, Matt Coffey, Peter Selby, Richard Vile, Alan Melcher

**Affiliations:** 1 Leeds Institute of Molecular Medicine, University of Leeds, Leeds, UK; 2 Institute of Cancer Research, Centre for Cell and Molecular Biology, Chester Beatty Laboratories, London, UK; 3 Postgraduate Medical School, University of Surrey, Guildford, UK; 4 Oncolytics Biotech Inc, Calgary, Alberta, Canada; 5 Molecular Medicine Program and Department of Immunology, Mayo Clinic, Rochester, Minnesota, USA

## Abstract

**Background:**

As well as inducing direct oncolysis, reovirus treatment of melanoma is associated with activation of innate and adaptive anti-tumour immune responses.

**Results:**

Here we characterise the effects of conditioned media from reovirus-infected, dying human melanoma cells (reoTCM), in the absence of live virus, to address the immune bystander potential of reovirus therapy. In addition to RANTES, IL-8, MIP-1α and MIP-1β, reovirus-infected melanoma cells secreted eotaxin, IP-10 and the type 1 interferon IFN-β. To address the mechanisms responsible for the inflammatory composition of reoTCM, we show that IL-8 and IFN-β secretion by reovirus-infected melanoma cells was associated with activation of NF-κB and decreased by pre-treatment with small molecule inhibitors of NF-κB and PKR; specific siRNA-mediated knockdown further confirmed a role for PKR. This pro-inflammatory milieu induced a chemotactic response in isolated natural killer (NK) cells, dendritic cells (DC) and anti-melanoma cytotoxic T cells (CTL). Following culture in reoTCM, NK cells upregulated CD69 expression and acquired greater lytic potential against tumour targets. Furthermore, melanoma cell-loaded DC cultured in reoTCM were more effective at priming adaptive anti-tumour immunity.

**Conclusions:**

These data demonstrate that the PKR- and NF-κB-dependent induction of pro-inflammatory molecules that accompanies reovirus-mediated killing can recruit and activate innate and adaptive effector cells, thus potentially altering the tumour microenvironment to support bystander immune-mediated therapy as well as direct viral oncolysis.

## Background

Reovirus is a nonenveloped dsRNA virus which is highly prevalent in the human population but produces few clinical symptoms. Great interest has surrounded the use of reovirus as an oncolytic agent due to its ability to infect and induce death in a range of human malignancies whilst sparing normal cells. Furthermore reovirus has completed a number of early clinical trials and is now being tested in the phase III setting [1-3]. Initial studies indicated that the tumour specific oncolytic activity was dependent upon the presence of an activated Ras signalling pathway [4], although recent data has indicated that susceptibility to reovirus infection may be influenced by additional complex mechanisms [5,6].

Previous work in our laboratory has indicated that human melanoma cell lines, as well as freshly resected tumour, undergo reovirus-induced apoptotic death in a Ras/RalGEF/p38 dependent manner, and that this death is accompanied by the release of inflammatory chemokines and cytokines [7]. The release of pro-inflammatory mediators following viral infection of tumour cells has been observed with other oncolytic viruses such as Herpes Simplex Virus (HSV) [8] and Newcastle disease virus (NDV) [9].

As well as inducing direct oncolysis, several viruses, either naturally or via insertion of immune-activating genes, have been shown to stimulate anti-tumour immune responses, indicating their potential as immunotherapeutic as well as cytotoxic agents [10]. We have previously shown that reovirus can exert immunogenic effects against tumour cells by directly activating DC to stimulate innate NK/T cell cytotoxicity [11], and by reovirus-induced tumour cell death facilitating the priming of innate and adaptive anti-tumour responses in mouse and human model systems [12-14]. However, the immunogenicity of the pro-inflammatory milieu produced by reovirus-infected melanoma cells (independent of the effects of the virus itself which may be cleared rapidly in vivo), and the signalling pathways involved in initiating cytokine/chemokine production in tumour cells, have not been addressed.

Chemokines can participate in the host response during infection and inflammation by directing immune effector cell migration. Four families of chemokines have been described based on the position of conserved cysteine residues [15]. Multiple chemokines can share one common receptor, and each chemokine can potentially bind to several different receptors, thereby allowing multiple biological outcomes depending upon the composition of the chemokine milieu and the cells within the environment [16,17]. Furthermore, at sites of inflammation, chemokines can form heteromers, potentially inducing synergistic actions and enhancing leukocyte migration and activation [18]. Hence, the induction of multiple chemokines within an immunosuppressive tumour microenvironment has the potential to induce potent effects on immune effector cells to enhance therapy. For example, in a murine B16 melanoma model, ectopic expression and secretion of IP-10 by tumour cells increased the number of NK cells at the tumour site and prolonged NK cell dependent survival [19]. Data have also indicated a good correlation between CXCR3 expression on T cells and an improved clinical outcome in stage III melanoma patients [20].

The current study further investigates the chemokines and cytokines (including type I IFNs) induced by reoviral oncolysis and the signaling pathways responsible for the production of these pro-inflammatory mediators. We also determine the effects of reoTCM, specifically in the absence of active virus to exclude the direct consequences of viral immune activation, on chemotaxis, activation and effector functions of NK cells, DC and CTL.

## Materials and methods

### Cell Culture and Reovirus

Skmel-28, Mel-624, Mel-888 and MeWo cells were grown in Dulbecco's Modified Eagle Medium (DMEM) (Invitrogen, Paisley, UK) supplemented with 10% (v/v) foetal calf serum (FCS) (BioSera, Crawley Down, UK) and 1% (v/v) L-glutamine (Sigma Aldrich). Peripheral blood mononuclear cells (PBMC) were obtained, with local ethics approval, from buffy coats of healthy blood donors by Ficoll-Hypaque density centrifugation. Human myeloid immature dendritic cells (DC) were generated from human PBMC by MACS CD14+ selection (Miltenyi Biotech) or monocyte adherence as previously described [21]. Monocytes were cultured in RPMI 1640 (Invitrogen Life Technologies) supplemented with 10% FCS, 1% L-glutamine (complete media), 800 U/ml GM-CSF (Schering-Plough) and 500 U/ml IL-4 (R&D systems) for 5 days. NK cells were isolated from human PBMC using MACS negative depletion kits following standard protocols as previously described (> 90% purity) [11]. NK cells were routinely cultured in DMEM supplemented with 10% (v/v) human AB serum (Sera Laboratories International Limited), 5% (v/v) foetal calf serum and 1% (v/v) L-glutamine. Cytotoxic T lymphocytes (CTL) primed using reovirus infected mel-888 cells (moi 0.1) were generated in a 14 day priming assay as detailed below. For chemotaxis assays, NK, DC and CTL were resuspended in RPMI/0.5%Human AB/1%L-Glutamine. All cell lines were grown at 37°C in an atmosphere containing 5% CO_2 _and were routinely tested for, and confirmed free of, *Mycoplasma*. Where indicated melanoma cells were treated with 2.5 mM 2-aminopurine (2-AP) (Sigma-Aldrich) or 50 μM caffeic acid phenethyl ester (CAPE) (Axxora, Nottingham, UK) for 1 hour prior to, and during, treatment with reovirus. Neither 2-AP nor CAPE at the doses used were directly toxic to melanoma cells (data not shown). Reovirus type 3 Dearing strain was provided by Oncolytics Biotech Inc. Virus titer was determined by a standard plaque assay using L929 cells.

### ELISA, Luminex and Flow Cytometry

IL-8 was detected using matched pair antibodies (BD Pharmingen). IFN-β was detected using an IFN-β kit (PBL Laboratories) as per the manufacturer's instructions. Eotaxin and IP-10 were detected using Luminex technology (Biosource) according to the manufacturer's instructions. To assess NK cell phenotypic activation, NK cells were labelled with CD69-APC/CD56-PE/CD3PerCP antibodies (BD Pharmingen, R and D Systems). CD69 expression was determined on gated CD56-PE^+ve^/CD3-PerCP^-ve ^populations using a FACSCalibur instrument (BD Biosciences).

### Western Blotting

Melanoma cell lines were seeded in 10 cm dishes and left untreated or infected with 10pfu/cell reovirus for 4, 8, 12, 16, 20 and 24 hours prior to preparation of whole cell lysates. Nuclear fractions were prepared using the Active Motif Nuclear Extract Kit according to the manufacturers' instructions. Whole cell lysates were prepared using RIPA buffer supplemented with a protease inhibitor cocktail (25 μl/ml, Sigma). 10 μg of total protein was loaded per lane for whole cell lysates. 20 μg of protein was loaded per lane for nuclear fractions. Proteins were separated on a 10% SDS polyacrylamide gel and transferred to a nitrocellulose membrane. Membranes were probed with antibodies against total PKR (BD Transduction Laboratories), I-κB (Santa Cruz) and p65 NF-κB (Upstate). Antibody binding was detected using the Odyssey system (LI-COR Biosciences UK, Cambridge, UK). Equal lane loading was confirmed using monoclonal antibody against β-actin (Sigma).

### RNA Interference (RNAi) Studies

siRNAs were purchased from Ambion and reconstituted to a concentration of 20 μM according to the manufacturers' instructions. An siRNA oligonucleotide against PKR (PKRV) and an irrelevant (green fluorescent protein-specific) control siRNA were used. The sequences were, PKRV 5' AAGGUGAAGGUAGAUCAAAGA-3', Control 5' AAGGACGACGGAAACUACAAG-3'. Melanoma cells in 24 well plates and 10 cm dishes were transfected with 100 nM (this concentration was optimized by initial titration experiments) of siRNA. Control siRNA transfections using equivalent concentrations were included in each experiment. Transfections were carried out in serum-free media using OligofectAMINE (Invitrogen) for 6 hours. Media were replenished with serum and supplements and cells were cultured in the continued presence of siRNA overnight. After the overnight incubation pre-existing media was removed from the 24 well plates and replaced with 1 ml normal growth media, and cells were left untreated or infected with reovirus (0-10pfu/cell). Supernatants from these cells were collected at 24 and 48 hours post reovirus infection. For 10 cm dishes the pre-existing media was removed and whole cell lysates were prepared and separated by SDS-PAGE prior to probing with total PKR antibody. Equal lane loading was confirmed using a monoclonal antibody against β-actin.

### Priming of Mel-888 Cytotoxic T Lymphocytes (CTL)

Mel-888 cells were seeded into tissue culture flasks and allowed to adhere. DC, resuspended in a 50:50 mix of DC media/reoTCM (from Mel-888), non-reoTCM, or DC media/DMEM complete, were added to the Mel-888 cells at a 1:3 ratio (DC:tumour cell) overnight. Suspension cells were aspirated, leaving the tumour monolayer intact, and cells were pelleted as previously described [13]. Tumour loaded DC were then resuspended in CTL media [RPMI supplemented with 7.5% (v/v) human AB serum (Sigma), 1% (v/v) L-glutamine, 1% (v/v) sodium pyruvate (Life Technologies), 1% (v/v) non-essential amino acids (Life Technologies), 1% (v/v) HEPES (Life Technologies), 20 μmol/L 2 -mercaptoethanol (Sigma), and mixed with autologous PBMC at a ratio of 1:10 to 1:30. Cultures were supplemented with 5 ng/ml IL-7 (R&D systems) from day 1 and 30 U/ml IL-2 (R&D systems) on day 4 only. Cultures were re-stimulated using the same protocol at day 7. Cells were harvested at day 14 and chromium release, CD107 degranulation and intracellular IFN-γ assessed as detailed below.

### NK, DC and CTL chemotaxis

1.5 × 10^5 ^melanoma cells were plated in 6 well plates and left overnight. Pre-existing media was removed and replaced with DMEM/2%FCS/1% L-Glutamine and cells were infected with 1 or 10pfu/cell reovirus for 48 hours. Supernatants were collected and passed through a 0.2 μm Acrodisc syringe filter (Pall Life Sciences) and then through a Viresolve^NFR ^filter (Millipore) to remove virus. Chemotaxis was assessed using a transwell system. 650 μl filtered TCM from each of the four melanoma cell lines was added to the lower chamber of triplicate wells in a 24-well plate and a 5 μm (NK cells and CTL) or 8 μm (DC) Thincert™ membrane (Greiner Bio-One) placed in the well. 5 × 10^5 ^NK, DC or CTL (the anti-Mel-888 CTL used in these chemotaxis experiments had been previously primed using reovirus-infected Mel-888-loaded DC as described [12]), resuspended in 100 μl of low serum media were placed in the upper chamber and plates were incubated at 37°C for 3 hours. Cells in the lower wells were harvested, washed in FACS buffer and labelled with 3 μl of CD56-PE (Serotec) and 3 μl CD3-FITC (BD Biosciences) (NK cells), CD11c-PE (BD Biosciences) (DC) or 3 μl CD3-FITC and CD8-PerCP (CTL) for 30 minutes at 4°C. Cells were washed, resuspended in 300 μl FACS buffer and transferred to a TruCount™ tube (BD Biosciences) containing a known number of fluorescent beads to provide the internal counting control. A cell to bead ratio was determined for each tube and a migration index was calculated by normalising this ratio to those of controls in which TCM harvested from uninfected tumour cells ('non-reoTCM') was used.

### CD107 degranulation and intracellular IFN-γ staining

CD107 is a marker of lysosomal granule exocytosis and was used as a marker of NK cell and CTL degranulation as previously described [14]. NK cells and CTL were incubated at a 1:1 ratio with tumour targets (K562, Mel-888 or SKOV-3), in the presence of CD107a-FITC and CD107b-FITC antibodies (BD Bioscience). Brefeldin A (10 μg/ml) was added after 1 hour and cells were incubated for a further 4 hours. Cells were then labelled with CD8 PerCP (for CTL) or CD56-PE, CD3-PerCP and Dead Cell Discriminator (Miltenyi Biotech) for NK cell cultures, and fixed in 1% PFA. Analysis was performed by gating on CD8 populations (CTL) or on CD56^+ve ^populations, and excluding cells labelled with Dead Cell Discriminator and CD3 in the FL3 channel (NK cells). For determination of intracellular IFN-γ, cells were treated as above, permeabilised with 0.3% saponin and labelled with IFN-γ-FITC (BD Pharmingen) prior to flow cytometric analysis.

### ^51^Chromium cytotoxicity assay

Cytotoxicity of CTL was measured using a standard 4 hour ^51^Cr assay [21] against Mel-888 or irrelevant SKOV-3 targets. Percent lysis was calculated using the formula: % lysis = 100 × (cpm experiment - cpm spontaneous release)/cpm maximum release - cpm spontaneous release.

## Results

### Reovirus-infected melanoma cells secrete eotaxin, IP-10 and IFN-β

We have previously shown that reovirus infection of human melanoma cell lines induces a potentially immunogenic form of cell death with the release of RANTES (CCL5), IL-8, MIP-1α (CCL3) and MIP-1β (CCL4) [7]. In addition, DC pulsed with reovirus-infected melanoma cells secrete the chemokines CCL2, 3, 4, 5, 7, 8, 11 (eotaxin), and CXCL10 (IP-10), and these culture supernatants induce NK cell chemotaxis [14]. We sought to extend these studies by first testing for secretion of this wider panel of chemokines from melanoma cell lines in response to direct reovirus infection. Having previously observed secretion of IFN-β (but not IFN-α) by DC pulsed with reovirus-infected Mel888 cells [14], we also tested for type 1 IFN production by reovirus-infected melanoma cells. Although the defective anti-viral IFN response of tumour cells to oncolytic viruses can account for tumour-specific oncolysis [22], this response may not be completely abrogated. IFN-β in particular may be functionally significant within the tumour microenvironment, since its secretion by reovirus-infected Mel888-loaded DC activates NK cells for enhanced cytotoxicity [14].

**Figure 1:**
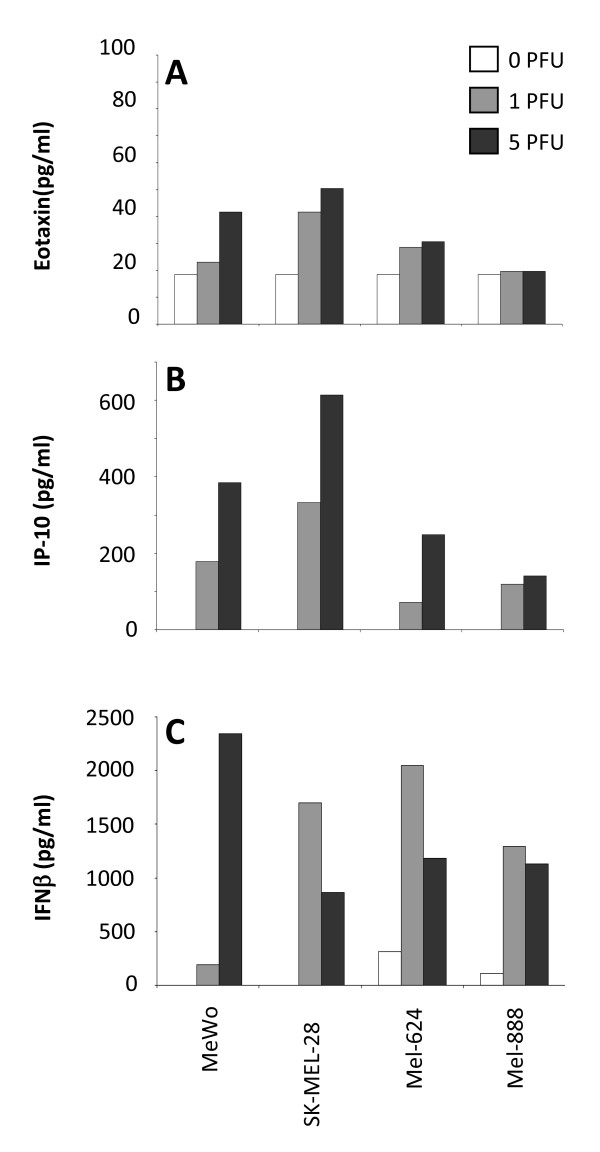
**Melanoma cell lines produce eotaxin, IP-10 and IFN-β in response to reovirus infection**. Melanoma cell lines were treated with 0 (open bars), 1 (pale grey bars) or 5 (dark bars) pfu/cell reovirus and supernatants were collected after 48 hours and assayed for eotaxin (A), IP-10 (B) and IFN-β (C). Results shown are representative of 3 independent experiments.

In addition to RANTES, MIP-1α, MIP-1β and IL-8 [7], we detected eotaxin and IP-10 (but not CCL2, 7 and 8 - data not shown) in all four cell lines tested. Levels of eotaxin were relatively low (Figure 1A), whilst IP-10 was secreted at higher levels by all cell lines (Figure 1B). Eotaxin is a member of the CC family of chemokines and can selectively recruit eosinophils [23], which have been associated with increased survival in a range of cancers [24]. IP-10 is a CXC chemokine, and a chemoattractant for monocytes, T lymphocytes and NK cells which has been shown to elicit immune-mediated anti-tumour effects *in vivo *[25].

Type 1 IFNs are secreted by normal cells to attenuate viral infections, and mediate multiple immunoregulatory functions that affect innate and adaptive responses [26], including phenotypic and functional maturation of DC [27] in the context of defence mechanisms against tumours [28]. Dysfunctional IFN pathways in cancer cells have been proposed as a mechanism by which replication and cell lysis for viruses such as vesicular stomatitis virus (VSV), vaccinia virus (VV), measles and NDV is restricted to tumour cells during oncolytic virotherapy [29]. Moreover, IFN-β has been genetically engineered into oncolytic viruses to improve the therapeutic index between normal and malignant cells [30], and to support priming of anti-tumour immunity [31]. Therefore, we tested whether type 1 IFNs were secreted by reovirus-infected melanoma cells, and found that IFN-β (Figure 1C), but not IFN-α (data not shown), was produced by all 4 cell lines. These data indicate that reovirus infection of melanoma cells induces inflammatory chemokines capable of recruiting immune effector cells, as well as IFN-β, which can support priming of anti-tumour immunity in the context of oncolytic virotherapy.

### Reovirus infection activates NF-κB in melanoma cells leading to chemokine/cytokine secretion

**Figure 2:**
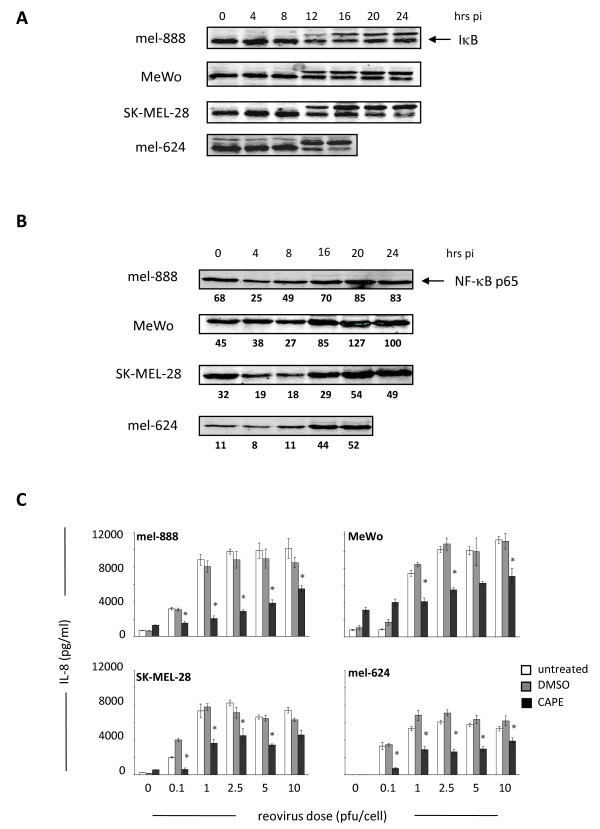
**Reovirus infection activates NF-κB in melanoma cells to induce cytokine secretion**. (A) Melanoma cell lines were seeded in 100 mm dishes and treated with 10pfu/cell reovirus. At 4, 8, 12, 16, 20 and 24 hours post-infection whole cell lysates were prepared and I-κB assessed by western blot. (B) Melanoma cell lines were seeded as in (A), and nuclear fractions were prepared and western blotted for NF-κB p65. Densitometry data is shown underneath each blot. (C) Melanoma lines were seeded in 24 well plates and pre-treated with 50 μM CAPE, or equivalent DMSO solvent concentrations, for 2 hours prior to addition of reovirus at the indicated doses. Supernatants were collected after 48 hours and IL-8 levels determined using ELISA. Data are representative of at least 3 independent experiments. * indicates *P *< 0.05, by Student's t-test.

Next, we investigated the signalling pathways involved in chemokine/cytokine production following reovirus infection of melanoma cells. We focused on NF-κB, as reovirus infection induces NF-κB nuclear translocation to activate pro-apoptotic gene expression in cultured HeLa cells [32]. Furthermore, several of the chemokines/cytokines produced in our system, such as IL-8, RANTES and IFN-β, are known NF-κB dependent genes [33]. NF-κB resides in an inactive cytoplasmic form in conjunction with I-κB. Following I-κB degradation, NF-κB translocates to the nucleus to initiate transcription. Therefore, I-κB degradation and increased expression of the p65 NF-κB subunit in nuclear fractions can be used as indirect indicators of NF-κB activation. In all 4 melanoma cell lines I-κB degradation was observed within 16 hours of reovirus infection, which coincided with an increase in nuclear p65 NF-κB expression (Figure 2A, B). To confirm a role for NF-κB, we used IL-8 and IFN-β as representative chemokines/cytokines, and pre-treated the melanoma cell lines with the NF-κB small molecule inhibitor CAPE [34] prior to infection with reovirus. In all cell lines pre-incubation with 50 μM CAPE led to significant decreases in IL-8 levels at all doses of reovirus used (p < 0.05) (Figure 2C). Similar inhibition of IFN-β secretion was observed following CAPE pre-treatment (data not shown). Taken together these data confirm that reovirus infection of melanoma cells induces NF-κB activation to initiate transcription of chemokines and cytokines.

### Chemokine and cytokine production by reovirus-infected melanoma cells is mediated by a PKR dependent pathway

**Figure 3:**
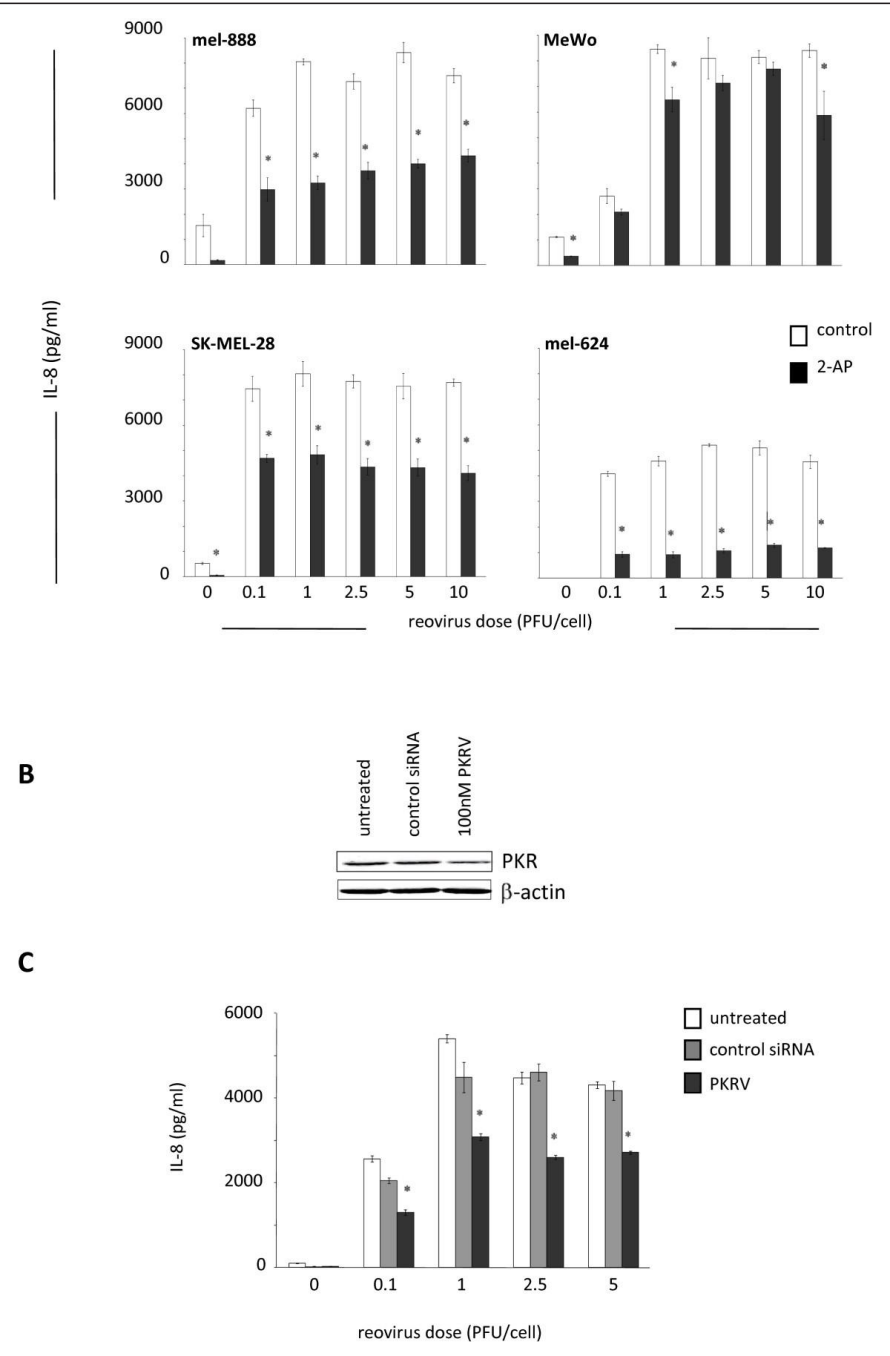
**PKR mediates cytokine production by reovirus treated melanoma cells**. (A) Melanoma cell lines were seeded in 24 well plates and pre-treated with 2.5 mM 2-AP or equivalent PBS controls, for 2 hours prior to addition of reovirus at the indicated doses. Supernatants were collected after 48 hours and IL-8 levels determined. Data are representative of at least 3 independent experiments. * indicates *P *< 0.05, by Student's t-test. (B) Mel-624 tumour cells were seeded in 100 mm dishes and transfected with 100 nM PKRV or irrelevant control siRNA. Cell lysates were prepared and western blotted for total PKR, with β-actin used to confirm equal track loading. (C) Mel-624 cells were seeded in 24 well plates and transfected with 100 nM PKRV or irrelevant control siRNA; reovirus was then added at the indicated doses. Supernatants were collected 24 and 48 hours later and IL-8 levels determined. Data are representative of two independent experiments. * indicates *P *< 0.05 by Student's t-test.

The double stranded RNA genome of reovirus is detectable by several cellular molecules which can activate multiple signalling pathways. Having established that reovirus infection induces NF-κB activation, we next sought to identify upstream mediators that might provide a link between dsRNA detection and NF-κB activation. A major candidate was the serine/threonine protein kinase PKR, which binds to, and is activated by, dsRNA. PKR can inhibit viral translation via phosphorylation of the translation initiation factor eIF-2α and ras-related defective PKR signaling has been implicated in the tumour specificity of reovirus replication and oncolysis [35]. PKR is also involved in the anti-viral type 1 IFN response, which is at least partially functional in our system, as demonstrated by secretion of IFN-β following reovirus infection (Figure 1C). Significantly, PKR is involved in the canonical NF-κB signalling transduction pathway [36], and can induce NF-κB activation via phosphorylation of I-κB [37]. We investigated the role of PKR in inflammatory chemokine/cytokine secretion by reovirus-infected melanoma cells, again using IL-8/IFN-β as representative readouts. Initial western blot analysis confirmed that all cell lines expressed baseline levels of total and phosphorylated PKR, which did not change on reovirus infection (data not shown). Cells were then pre-treated with the PKR inhibitor 2-AP prior to reovirus infection and significant reductions in IL-8 (p < 0.05) (Figure 3A) and IFN-β (data not shown) were observed in 3 out of 4 cell lines. To further confirm these findings we used siRNA to specifically knockdown PKR expression [38]. Mel-624 cells were used, following initial optimization studies, as these were found to have the highest transfection efficiency of the 4 cell lines (data not shown). PKR siRNA decreased total PKR expression by approximately 50% compared with control siRNA treated cells (Figure 3B). This knockdown was found to correlate to an approximate 40% reduction in IL-8 secretion 24 hours post reovirus infection (p < 0.05) (Figure 3C). These data confirm a role for PKR, in addition to NF-κB, in induction of inflammatory chemokines/cytokines upon reovirus infection and oncolysis.

### Virus-filtered tumour conditioned media from reovirus-treated melanoma cells (reoTCM) induces a chemotactic response in NK cells, DC and CTL

Previously identified components of reoTCM, such as MIP-1α, MIP-1β, RANTES, [7], Figure 1B) are chemoattractants to a variety of immune cell types. We tested whether reoTCM could induce a chemotactic response in relevant immune effector cells (NK, DC, CTL). To address the potential immunogenic bystander effects of the chemokines and cytokines independent of direct consequences of the virus itself [11], reoTCM was passed through a Viresolve^NFR ^filter, and successful removal of reovirus was confirmed by a negative plaque assay on L929 cells (data not shown).

**Figure 4:**
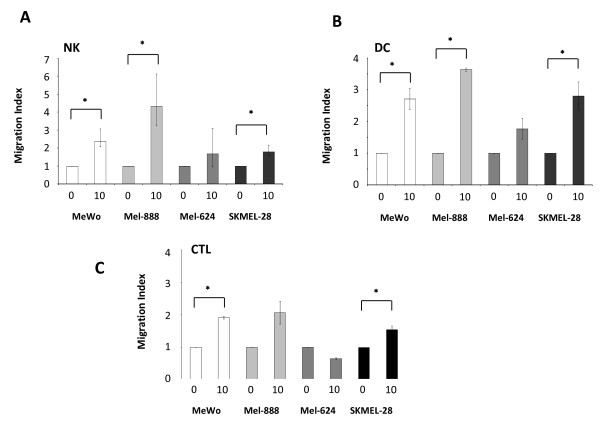
**Isolated NK cells, DC and CTL migrate toward virus filtered reoTCM**. 5 × 10^5 ^NK cells (A), DC (B) or CTL (C) were resuspended in RPMI + 0.5% human AB serum + 1% L-Glutamine and placed in a 5 μm (NK + CTL) or 8 μm (DC) Thincerts™ to separate cells from virus filtered reoTCM/non-reoTCM. Migration was assessed after 3 hours by labelling cells with CD11c-PE (DC), CD56-PE/CD3-FITC (NK cells) or CD3-FITC/CD8-PerCP (CTL) and then using Trucount™ tubes to provide an internal counting control. A cell:bead ratio was determined for each tube and a migration index calculated by normalising this ratio to those of non-reoTCM controls. Data represents means of triplicate wells +/- SEM and is representative of 4 independent donors. * indicates *P *< 0.05 by Student's t-test.

Isolated NK cells actively migrated toward reoTCM (Figure 4A), with an approximate 2-3 fold increase in migration in 3 out of 4 cell lines (p < 0.05). Similar chemotactic responses were observed in DC with reoTCM derived from the same three cell lines (p < 0.05) (Figure 4B). Anti-tumour effector CTL generated by priming in the presence of reo-infected mel-888 [12] gave comparable results (Figure 4C); increases in cell migration were observed for MeWo, SKMEL-28 (p < 0.05) and Mel-888 (although this did not reach statistical significance). Mel-624 reoTCM failed to induce a significant chemotactic response in any cell type. Interestingly, this cell line exhibited lower levels of MIP-1α, MIP-1β and IP-10 secretion following reovirus infection ([7], Figure 1B). Although these data do not precisely define the single/multiple chemokines responsible for chemotaxis, they show that NK cells, DC and CTL are capable of actively migrating toward melanoma cells undergoing reoviral induced cell death.

### ReoTCM from reovirus-treated melanoma cells induces phenotypic and functional activation of NK cells

**Figure 5:**
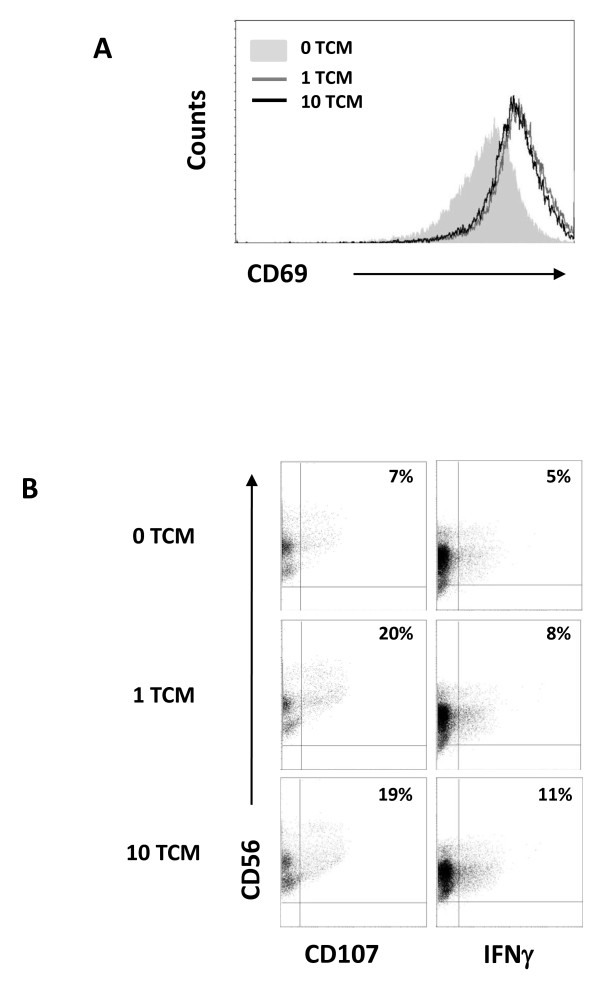
**NK cells cultured in reoTCM are activated against melanoma cell targets**. (A) NK cells were cultured in the presence of virus-filtered reoTCM/non-reoTCM overnight and CD69 expression determined. (B) NK cells were cultured in the presence of filtered reoTCM or non-reoTCM for 48 hours and then co-cultured with Mel-888 tumour targets prior to CD107 and intracellular IFNγ assessment. Results shown are gated on CD56^+ve^/CD3^-ve ^cell populations and are representative of 4 independent donors.

Previous work in our laboratory has demonstrated that secretion of IFN-β by DC loaded with reovirus infected Mel-888 cells stimulated NK cell cytotoxicity toward melanoma targets [14]. Having demonstrated that reoTCM contains IFN-β and that virus-free reoTCM can be chemoattractant to immune effector cells (Figure 1C, 4A), we investigated the immunogenic potential of reoTCM with regard to NK cell activation and innate anti-tumour immune priming. Culture of isolated NK cells in Mel-888 reoTCM upregulated the expression of the NK activation marker CD69 (Figure 5A) and increased levels of NK degranulation and intracellular IFN-γ (Figure 5B) following co-culture with Mel-888 targets. ReoTCM-treated NK cell degranulation was also demonstrated against K562 targets (although not in the absence of targets - data not shown) demonstrating the non-specific nature of this innate response. Hence, the pro-inflammatory environment induced by reovirus infection of melanoma cells is capable, even in the absence of replicating virus, of inducing lytic activity and intracellular IFN-γ in activated NK cells, thereby potentially supporting innate anti-tumour effects within a treated tumour.

### Tumour cell-pulsed dendritic cells cultured in reoTCM prime human naïve anti-melanoma CTL

**Figure 6:**
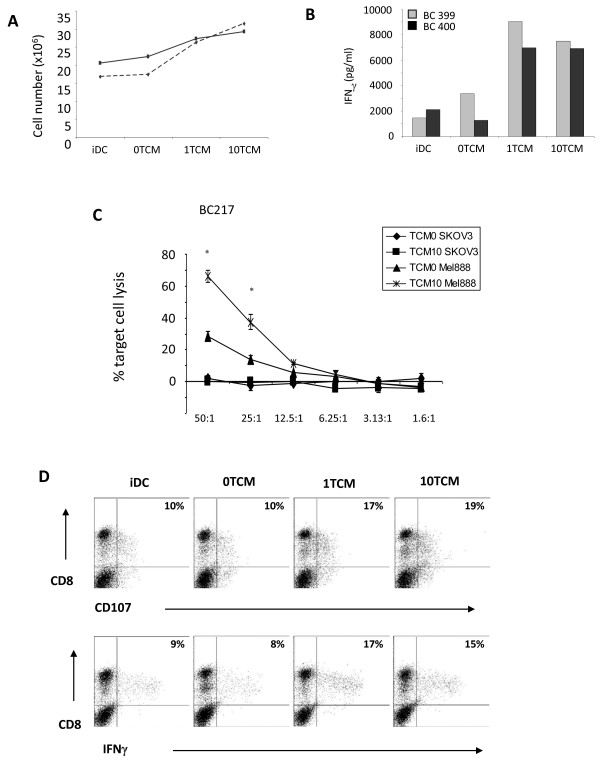
**ReoTCM effectively supports priming of specific CTL by tumour cell-loaded DC**. PBMC were incubated with autologous DC that had been cultured overnight with Mel-888 tumour cells in the presence of reoTCM/non-reoTCM. The PBMC were restimulated 7 days later and assayed at 14 days. (A) Lymphocyte proliferation was determined via trypan blue exclusion (2 representative donors are shown). (B) IFN-γ levels in CTL supernatants were determined by ELISA (2 representative donors are shown). (C) Cytotoxicity of lymphocytes primed in the presence of reoTCM versus non-reoTCM was determined by ^51^Cr release assay using Mel-888 tumour cells as specific targets and SKOV-3 as irrelevant controls. One donor is shown as representative of 2 independent experiments. * indicates *P *< 0.05 by Student's t-test. (D) CTL as in (C) were further assayed for CD107 degranulation and intracellular IFN-γ on co-culture with Mel-888 targets. The results of one donor are shown, representative of at least four independent experiments.

We have previously shown that reoTCM can induce phenotypic maturation of DC [11], and that DC loaded with infected Mel-888 cells can prime tumour specific CTL in the continued presence of reovirus [12]. To address the adaptive immunogenic potential of virus-free reoTCM, DC were cultured in reoTCM (or medium from uninfected non-reoTCM controls), loaded with Mel-888 cells and added to PBMC. The generation of anti-tumour CTL over 14 days was then assessed as previously described [13]. PBMC proliferation was measured using trypan blue exclusion to determine viable cell number and was greater in reoTCM priming cultures than non-reoTCM controls (Figure 6A). Moreover, this proliferation was associated with increased levels of IFN-γ in the priming culture supernatants, consistent with an evolving Th1 adaptive T cell response (Figure 6B). A chromium cytotoxicity assay was used to determine the lytic ability of CTL generated by reoTCM and non-reoTCM-treated tumour-loaded DC. Whilst some specific anti-Mel888 CTL activity was seen under non-reoTCM DC conditions, levels of killing were significantly higher when reoTCM-conditioned DC were used for CTL priming (approximately 70% lysis compared with 30%) (p < 0.05) (Figure 6C). No killing of irrelevant SKOV-3 tumour targets was observed. In addition, CTL CD107 degranulation and intracellular IFN-γ, in the presence of Mel888 targets (but not SKOV-3, data not shown), was also higher after reoTCM priming compared with their non-reoTCM counterparts (Figure 6D). Although these cytotoxicity assays do not address the MHC class I restriction or antigen specificity of killing (which are potential confounding factors when using an allogeneic tumour cell line as an antigen source for human CTL priming), we have previously shown in this system that specific anti-Mel888 CTL include T cells which recognize the tumour-associated antigen MART-1 [12,13], demonstrating that these responses include targeting of antigens relevant to anti-tumour therapy. Hence, in addition to activation of innate NK cell anti-melanoma activity, virus-free reoTCM is able to support effective priming of a specific adaptive CTL response.

## Discussion

Reovirus is a tumour-specific oncolytic virus currently under clinical investigation [39,40]. We, and others, have shown that reovirus is one of several therapeutic viruses whose activity can be mediated via activation of an anti-tumour immune response, as well as the direct oncolytic effect of viral replication in tumour cells [10]. Whether the immune response to viral therapy is problematic, due to rapid systemic inactivation of the agent, or actively therapeutic, via provision of a 'danger' signal within an otherwise immunosuppressive tumour microenvironment, likely depends on multiple factors. These include route of virus delivery (intratumoural *versus *intravenous), the pre-existing immune status of the patient and the mechanisms by which the virus naturally, or via genetic modification, targets tumour cells. Consequently, various immunomodulatory strategies have been employed to improve oncolytic viral therapy, ranging from immunosuppression to improve viral persistence in the circulation [41], through to enhancement of immune activation via insertion of transgenes, such as GMCSF, into the viral genome [42].

We have previously shown that i) reovirus induces apoptotic death in human melanoma cells and that this death is associated with secretion of inflammatory chemokines/cytokines [7], ii) reovirus directly activates DC in the absence of tumour cells [11], and iii) reovirus-infected melanoma cells can activate innate and adaptive arms of the anti-tumour immune response [12-14]. However, these data were generated in the continued presence of active virus. Since reovirus itself is directly immunostimulatory [11], removal of the virus from reoTCM via filtration allowed us to specifically investigate the additional, bystander immunogenic effects of the inflammatory environment potentially generated in treated tumours. The immunogenic component of human reoviral therapy may have particular clinical relevance, since levels of reovirus replication in freshly resected melanoma cells may be low [7]. Furthermore, the switch from a suppressive to an inflammatory tumour milieu may persist even after the virus has been cleared [43].

We extended our previous analysis of the chemokines and cytokines produced by reovirus-infected human melanoma lines, and showed that eotaxin and IP-10 were also secreted (Figure 1). Interestingly, we also detected IFN-β (but not IFN-α) under these conditions, illustrating that an anti-viral type 1 IFN response is partially functional in these tumour cells. This is particularly important as IFN-β is involved in innate immune activation by DC loaded with reovirus-infected cells [14]. Moreover, IFN-β has been engineered into other oncolytic viruses to increase the therapeutic index between malignant and normal cells, and to enhance anti-tumour immune activation [30].

Although the mechanisms responsible for the inflammatory response of tumour cells to reovirus infection have not been addressed to date, previous studies have implicated a role for NF-κB since i) reovirus infection initiates translocation of the p50/p65 NF-κB subunits to the nucleus and activates pro-apoptotic gene expression [32,44], ii) reovirus induces apoptosis in melanoma cells [7], and iii) NF-κB is involved in the production of chemokines and cytokines such as IL-8 and IFN-β [33]. This study confirms that reovirus infection of melanoma cells activates NF-κB, as assessed by I-κB degradation and accumulation of nuclear p65, and that blocking NF-κB with the small molecule inhibitor CAPE significantly decreases production of IL-8 and IFN-β (Figure 2). Importantly, this effect was seen across all 4 cell lines, suggesting that common signalling pathways are activated following reovirus infection of melanoma. To address viral sensing and signaling molecules that may lie upstream of NF-κB, we investigated the dependence of IL-8 and IFN-β production on PKR, as one of a number of candidate dsRNA sensors. PKR is involved in the tumour specificity of reoviral oncolysis (although the precise mechanism remains to be fully elucidated), and the anti-viral type 1 IFN response [35]. We found, via small molecule blockade and siRNA knockdown, that PKR is involved in the inflammatory response of melanoma cells following reovirus infection (Figure 3). Although we have been unable to detect any significant change in total or phosphorylated PKR following reovirus infection of melanoma cell lines (data not shown), these data suggest that dsRNA detection by PKR initiates activation of NF-κB-dependent chemokines and cytokines in tumour cells. These findings are in agreement with previous observations following direct infection of DC [11]. However, further work is required to fully characterize the signaling pathways responsible for the immunogenic nature of reovirus-induced tumour cell oncolysis.

Filtered reoTCM induced a chemotactic response in NK cells, DC and anti-tumour CTL (previously primed using reovirus-infected tumour cells) (Figure 4), suggesting that the immunogenic milieu in treated tumours has the potential to recruit a range of immune cells capable of viral detection and innate/adaptive effector functions. With regard to improving access of primed CTL to tumours, this finding is consistent with previous murine data showing greater persistence of adoptively transferred antigen specific T cells within tumours undergoing VSV-mediated oncolysis [45]. To date we have not specifically identified which of the secreted chemokine(s) are responsible for NK cell, DC and CTL migration. It is possible that multiple chemokines may act in combination to engage a variety of receptors to induce a particular physiological response [16]. The ability of reoTCM to support activation of innate (Figure 5) and adaptive (Figure 6) immune responses against human melanoma cells shows that the immunogenic effects of reovirus induced cell death are not dependent on the continued presence of virus once an initiating danger signal has been delivered. Therefore, even if viral replication in patients is limited by neutralization, for example following repeated administration [46], the immunogenic response of tumour cells to reovirus infection may be sufficient to induce continuing anti-tumour effects.

Overall, the present study shows that reovirus infection of human melanoma cells induces a range of chemokines and cytokines capable of inducing a chemotactic response in NK cells, DC and primed CTL. This inflammatory response is dependent upon NF-κB and PKR and is sufficient, in the absence of live virus, to support priming of innate and adaptive anti-tumour immunity. This data supports the potential of bystander activation of human anti-tumour immunity by reovirus killing of tumour cells, even if persistent viral replication is limited by the anti-viral immune response.

## Abbreviations

TCM: Tumour conditioned media; RANTES: regulated on activation normal T expressed and secreted; MIP: macrophage inflammatory protein; IFN: Interferon; PKR: Protein kinase R; NK: Natural Killer; DC: dendritic cells; CTL: cytotoxic T lymphocytes; NDV: Newcastle Disease Virus; HSV: Herpes Simplex Virus; DMEM: Dulbecco Modified Eagle Medium; FCS: Foetal Calf Serum; PBMC: peripleral blood mononuclear cells; CAPE: caffeic acid phenethyl ester; 2-AP: 2-aminopurine; FACS: fluorescent activated cell sorting; VSV: vesicular stomatitis virus; GMCSF: granulocyte macrophage colony-stimulating factor; siRNA: small interfering RNA; SDS-PAGE: sodium dodecyl sulphate polyacrylamide gel electrophoresis;

## Competing interests

Oncolytics Biotech Inc: KH/RV/HP/AM, commercial research grant. MC, employee.

## Authors' contributions

LS contributed to conception and design, acquisition of data, analysis and interpretation of data and wrote the manuscript. FE, RP, HP, KH, PS, RV and EI contributed to conception, analysis and interpretation of data (as did MC who also provided clinical grade reovirus (Reolysin). AM conceived the study, participated in its design and coordination and co-wrote the manuscript. All authors read and approved the final manuscript.
